# Supplementary material for: Exploratory Analyses of Circulating Neoplastic-Immune Hybrid Cells as Prognostic Biomarkers in Advanced Intrahepatic Cholangiocarcinoma
Source: Int J Mol Sci. 2024 Aug 24;25(17):9198. doi: 10.3390/ijms25179198 (PMC11395231; doi:10.3390/ijms25179198)
Supplement: Supplementary file 1 [file ijms-25-09198-s001.zip › ijms-3156809-supplementary.pdf]

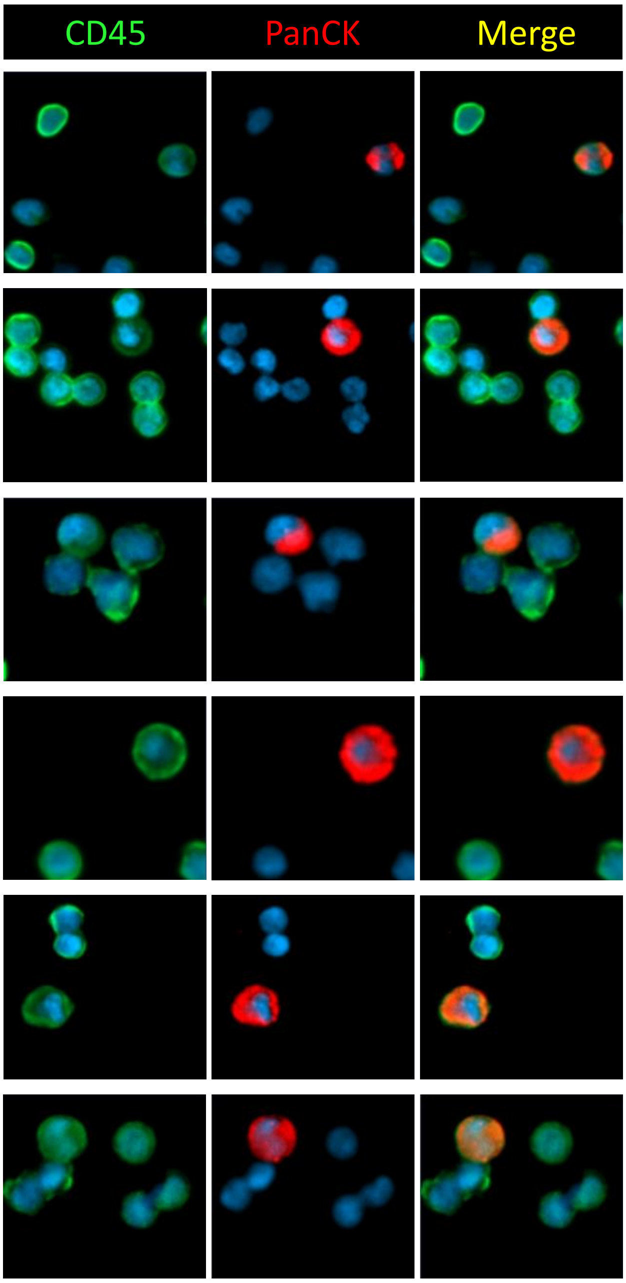

Figure S1. Representative CK$^+$/CD45$^+$ circulating hybrid cells (CHCs) from the entire study cohort.

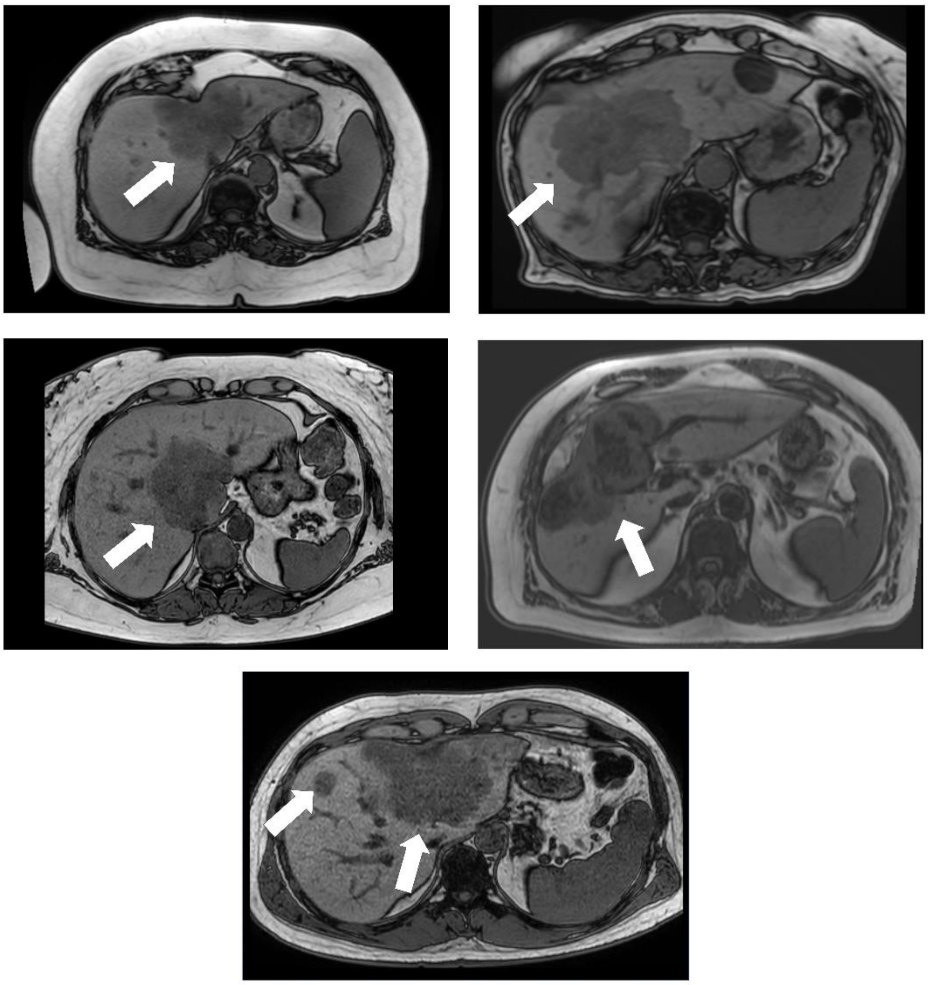

Figure S2. Representative cross-sectional images from study cohort. White arrows denote visualized intrahepatic cholangiocarcinoma lesions.

Supplemental Table S1. Clinical and Treatment Characteristics per Patient.

Patient	1	2	3	4	5
Median baseline CA 19–9, U/mL	830.3	16.8	6.3	20.5	140.5
Median baseline CHC count, per 50,000 PBMCs	13.5	14.7	15.5	8.4	7.6
Systemic therapy prior to trial enrollment	Yes, Gem/Cis, 1 cycle	No	No	No	No
Dominant lesion size, cm.	8.7	9.8	8.4	14.5	11.2
Number of intrahepatic lesions, no.	9	1	1	14	15
Large vessel involvement	Yes	Yes	Yes	Yes	Yes
Follow up from trial enrollment, months	6.0	27.5	20.1	25.6	22.7
Number of blood samples collected per patient, no.	9	7	9	7	9
Number of blood samples collected per patient while on trial protocol treatment, no.	7	6	7	7	6
Number of blood samples collected per patient after completion of trial protocol treatment, no.	0	3	3	2	1

Abbreviations: CA 19–9, carbohydrate antigen 19–9; CHC, circulating hybrid cell; no., number; Gem/Cis, gemcitabine and cisplatin.
